# The GReat-Child™ Trial: A Quasi-Experimental Intervention on Whole Grains with Healthy Balanced Diet to Manage Childhood Obesity in Kuala Lumpur, Malaysia

**DOI:** 10.3390/nu10020156

**Published:** 2018-01-30

**Authors:** Hui Chin Koo, Bee Koon Poh, Ruzita Abd Talib

**Affiliations:** 1 Nutritional Sciences Programme, School of Healthcare Sciences, Faculty of Health Sciences, Universiti Kebangsaan Malaysia, 50300 Kuala Lumpur, Malaysia; hckoo@tarc.edu.my (H.C.K.); pbkoon@ukm.edu.my (B.K.P.); 2 Department of Bioscience, Faculty of Applied Sciences, Tunku Abdul Rahman University College, 50300 Kuala Lumpur, Malaysia

**Keywords:** childhood obesity, intervention, Malaysia, whole grain

## Abstract

**Background:** The GReat-Child Trial was a quasi-experimental intervention that has emphasized whole grain as a strategy to manage childhood obesity. **Methods:** Two schools in Kuala Lumpur with similar demographic characteristics were assigned as intervention (IG) and control (CG). Eligibility criteria were overweight/obese children aged 9 to 11 years who had no serious co-morbidity. Children who reported consuming wholegrain foods in their 3-day diet-recall during screening were excluded. A total of 63 children (31 IG; 32 CG) completed the entire intervention program. The IG children underwent six 30-min nutrition education lessons and had school delivery of wholegrain food on a daily basis over a 12-week period. Parents of IG children attended 1-h individual diet counseling. Anthropometric outcomes including BMI-for-age z-score (BAZ), body fat percentage and waist circumference were measured at baseline [T0], post-intervention [T1] (3rd month) and follow-up [T2] (9th month). **Results:** IG showed significantly lower BAZ (weighted difference: −0.12; 95% CI: −0.21, −0.03; *p* = 0.009), body fat percentage (weighted difference: −2.6%; 95% CI: −3.7, −1.5; *p* < 0.001) and waist circumference (weighted difference: −2.4 cm; 95% CI: −3.8, −1.0; *p* = 0.001) compared to CG. IG reported significantly lower body fat percentage (weighted difference: −3.4%; 95% CI: 1.8, 5.0; *p* < 0.001) and waist circumference (weighted difference: −2.1 cm; 95% CI: −3.7, −0.5; *p* = 0.014) at T1 compared to T0. **Conclusions:** The GReat-Child Trial made a positive impact in managing childhood obesity. It can be incorporated into childhood obesity intervention programs that are being implemented by the policy makers.

## 1. Introduction

Childhood obesity is increasing at an alarming rate throughout the world. A consistent increase in the prevalence of childhood obesity has been observed since 1971 in developed countries; unfortunately, its prevalence is increasing in developing countries as well [1]. In Malaysia, the Ministry of Health carried out the third National Health and Morbidity Survey (NHMS) in 2006 and found that 5.4% of children younger than 18 years of age were overweight/obese [2]. Nine years later, the NHMS revealed that the prevalence of childhood obesity had increased to 11.9% [3]. Childhood obesity may be increasing at a faster rate than adult obesity and prompts the development of biomarkers for serious illnesses later in life [4]. The long-term persistence of obesity in childhood is associated with several health hazards, such as fatty liver disease, diabetes and cardiovascular diseases [5]. There are several factors that contribute to the childhood obesity epidemic. For a large majority of individuals, childhood obesity results from excess calorie consumption [6]. Unhealthy eating patterns contribute in a major way toward development of childhood obesity.

Public health researchers and clinicians agreed that primary and secondary prevention could be the mainstay plan for controlling the current epidemic of childhood obesity. Commonly suggested modifiable dietary habits strategies to combat childhood obesity include encouraging breastfeeding, increasing fruit and vegetable intake, increasing whole grains intake, limiting sweetened drink consumption and controlling portion sizes [7]. It is increasingly recognized that both clinical interventions and supportive institutional and community environments are required for individuals to adopt healthier eating pattern to manage childhood obesity [8]. Several childhood obesity interventions testing nutritional education and diet modification have been initiated in several countries, such as Malaysia [9], Singapore [10], New Zealand [11] and the United States of America [12]; however, none of these interventions emphasized the provision of whole grains to manage childhood obesity.

Intake of whole grains among children had been intensively studied. To date, globally accepted definition of whole grain does not exist; however, the American Association of Cereal Chemists International (AACCI) definition has been widely adopted. The AACCI defines a whole grain as consisting of the cracked, ground, intact or flaked caryopsis, whose principal anatomical components, the germ, bran and starchy endosperm are present in the same relative proportions as they exist in the intact grain [13]. Substantial scientific evidence suggested a negative association between whole grains consumption and the risk of childhood obesity [14]. Whole grains have lower energy density and together with its physico-chemical properties such as viscosity and bulking properties, which is good for weight maintenance [15]. The Malaysian Dietary Guidelines for Children and Adolescents recommends four to nine servings of grains’ foods per day according to age and emphasized that at least half of the grain servings should be from whole grains sources [16]. A meta-analysis concluded that individuals consuming more than three servings of wholegrain foods had consistently lower risk of obesity [17]. However, many national dietary surveys found that intake of whole grains in children are way lower than the recommendation [18,19,20]. A high whole grains intake would be desirable in early life since food preferences are shaped at an early stage in life and can be tracked until adulthood [21].

A quasi-experimental trial that aimed to increase whole grains intake among children was conducted in the United States; however, this trial did not investigate the effect of whole grains intervention in managing childhood obesity [22]. Thus, the GReat-Child Trial was carried out with the aim of emphasizing the whole grains as a strategy to improve anthropometric measurements of overweight/obese children. To the best of our knowledge, this is the first trial that has focused on whole grains to manage childhood obesity. The aim of the present trial was therefore to test the hypothesis that a whole grains intervention for the treatment of childhood obesity would have a greater effect on anthropometric measurements.

## 2. Methodology

### 2.1. Study Design and Participants

The overview, rationale, trial design and methods used in the GReat-Child Trial have been described in detail elsewhere [23]. In brief, the GReat-Child Trial was a 12-week quasi-experimental intervention with a 6-month follow up. It consisted of an intervention school and a control school from Keramat zone in Kuala Lumpur, Malaysia. This trial consists of three components addressing the behavior, personal and environmental factors based on the Social Cognitive Theory: (1) six 30-min nutrition education classes, which employed Food Guide Pyramid and visual plate model in emphasizing the whole grains recommendation and balanced diet; (2) 12-week school delivery of whole-grain foods which consisted of whole-grain bread, whole-grain biscuits and whole-grain ready-to-eat cereal, on a daily basis to provide opportunity for the children to experience and accept eating wholegrain food; and (3) a family involvement component where a parent attended an hour of individual diet counseling to encourage them to increase the availability of wholegrain food and to practice a balanced diet at home.

The inclusion criteria were: (1) apparently healthy Malaysian schoolchildren aged 9–11 years and studying in Year 4 or Year 5; (2) children who were overweight or obese; (3) able to read, write and understand Malay and (4) at least one parent who perceived that their child has a weight problem and who is willing to attend the individual diet counseling. Children were excluded if: (1) they have a serious co-morbidity requiring treatment or were on gluten-free diet and (2) any of their three-day 24-h diet-recall during screening indicated that they had consumed whole-grain food.

The study protocol was reviewed and approved by the University Kebangsaan Malaysia Research Ethics Committee and permission to carry out data collection was granted by the Ministry of Education, Malaysia and the Kuala Lumpur Federal Territory Education Department. Parental consent was obtained for all children prior to participation, while verbal assent was also obtained from the children before the study began.

### 2.2. Sample Size Calculation

The sample size was estimated using Naing’s (2009) equation [24]. The standard deviation (SD) of BMI-for-age z-score (BAZ) from a previous study was taken into consideration for the sample size calculation of the GReat-Child trial [25]. With the SD of the BAZ equal to 0.10, giving a detectable difference of 0.08, a sample size of around 25 children per group at 12 weeks would give 80% power at the 0.05 significance level.

n = [2σ^2^/∆^2^]·(Z_α_ + Z_β_)^2^

where,

n = estimated sample size
σ = standard deviation for BMI-score from previous study = 0.10
∆ = detectable difference = 0.08
Z_α_ = significance level for two-sided test = 1.96
Z_β_ = 80% power of study = 0.84

The sample size required for this trial was 25 per group. While taking into account a non-response rate of 50%, the required sample size for each group was increased to 38; therefore, the total number of children needed was 76. Children were allowed to dropout from the trial, with the reasons being noted and analyzed.

### 2.3. Anthropometric Measurements

Anthropometric outcomes were assessed on three occasions, at the baseline (T0), at the thirteenth week (T1; after 12-week intervention) and at the ninth month (T2; after 6-month follow-up), which were compared between the intervention and control groups. All assessments were done by a single investigator throughout the study to avoid inter-interviewer variations.

Body weight and height of each child were measured twice, according to standard procedures, using a calibrated Tanita digital scale Model SC-330 (Tanita Co., Tokyo, Japan) and SECA Bodymeter 217 (SECA GmbH & Co., Hamburg, Germany), respectively. The measurements were recorded to the nearest 0.1 kg and 0.1 cm, respectively. The anthropometric status of the children was determined based on the World Health Organization (WHO) growth reference for children aged 5–19 years old [26]. BAZ was determined using the WHO AnthroPlus version 1.0.3 (World Health Organization, Geneva, Switzerland) software and children were classified into overweight (BAZ +1SD to +2SD) and obese (BAZ > +2SD) categories.

The percentage of body fat was measured twice by bioelectrical impedance using the TANITA digital scale Model SC-330 (Tanita Co., Tokyo, Japan) to the nearest 0.1%. The children stood bare-foot on the footpads as the shapes of electrodes guide, after the electrode of the scale was cleaned to remove any debris. Any objects that could interfere with the readings were removed from their pockets before the measurements were taken.

Waist circumference (WC) of the children was measured twice, according to the standardized protocol by the WHO (2008) [27], which measured the WC at the approximate midpoint between the lower margin of the last palpable rib and the top of the iliac crest. The WC was measured to the nearest 0.1 cm by using a Lufkin tape model W606PM (Apex Tool Group, Sparks, MD, USA). Finally, the reported values of the body weight, height, percentage of body fat and WC were the average values from the two readings taken.

### 2.4. Physical Activity Measurement

In the GReat-Child Trial, physical activity was measured using a pedometer, Digi-walker CW-701 (Yamax, Fukuyama, Japan), which measures step-counts during the children’s waking hours over seven consecutive days for the intervention and control groups. The children were instructed to wear the pedometer on a waist belt, at all times, except while sleeping, swimming and showering. The pedometer has a memory recall to allow the investigator to recover the step-counts and weighted average daily step-counts were calculated from the weekdays and weekend days. A minimum number of valid days (at least 3 weekdays and 1 weekend day) were used for the calculation of average pedometer step-counts [28]. The pedometer step-counts were considered valid if the weighted average step-count are more than 1000 steps per day [29] and the children wore the pedometer for at least 10 h per day [28].

### 2.5. Statistical Analyses

Statistical analysis was conducted using Statistical Package for the Social Sciences (SPSS) version 22.0 (IBM SPSS Statistics 2014, IBM Corp., Armonk, NY, USA). Data were entered, cleaned and checked before data analyses. Each variable was examined for normality distribution using the Shapiro-Wilk test. Categorical data were presented as number and percentage. Continuous data were presented as mean and standard deviation (SD). The differences between intervention and control groups across socio-demographic data were determined using chi-square test. The differences between intervention and control group across anthropometric measurements and pedometer step-counts were determined using independent *T*-test. Analysis of covariance for repeated measures (ANCOVA) was performed to determine the GReat-Child trial effects. Two models were examined, including (1) within group differences and (2) between groups’ differences. Baseline variables were considered as covariates in each model in order to prevent bias [30]. Model assumptions including normality of the residuals, homogeneity of variance, compound symmetry and homogeneity of regression were verified. *p*-value of less than 0.05 for a two-sided test was considered statistically significant.

## 3. Results

### 3.1. Flow of Participants through the Trial and Their Characteristics

**Figure 1:**
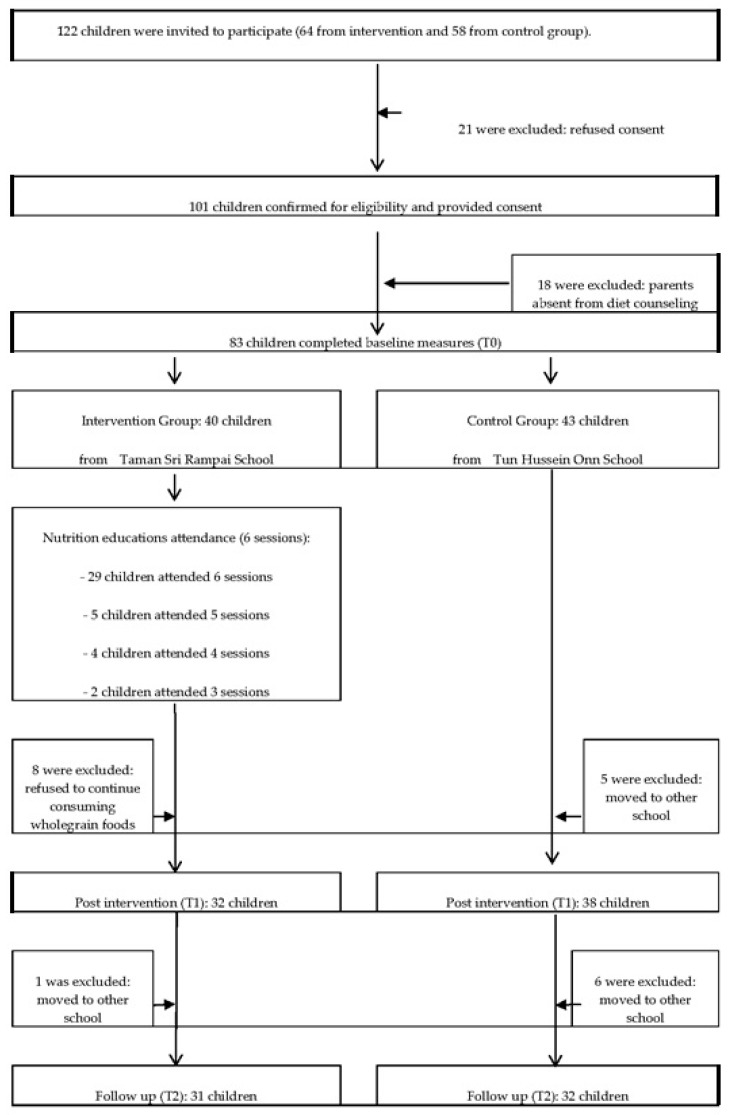
Flow of participants through the GReat-Child trial.

Figure 1 describes the flow of participants through the trial. A total of 122 potential children within the inclusion criteria were invited to participate in the GReat-Child Trial (64 from intervention group; 58 from control group) and 101 agreed to take part, resulting in a response rate of 82.7%. Of the 101 children who agreed to take part, 18 parents were absent from diet counseling, 12 children had moved to other schools and a further 8 children refused to continue consuming wholegrain foods, resulting in a final sample size of 63 children at T2.

A summary of 63 overweight/obese children’s baseline demographic characteristics, anthropometric measurements and pedometer step-count are shown in Table 1. A total of 31 parent/child pairs in the intervention group and 32 children in the control group participated and successfully completed the entire trial. Median age of the children was 10.5 (IqR 1) years, with approximately 1:1.1 girl and boy sex ratio. A majority of the children were belonged to the medium household income family (79.4%), with earning ranged from RM2300 to RM5599 per month, based on the classification by the Malaysian Economic Planning Unit (2010). Mean BAZ of the children was categorized in the obese group. All the anthropometric measurements data were available for all the study participants. However, for the pedometer step-counts, 8 data points (12.7%) were missing due to poor compliance with the pedometer protocol. At baseline, the intervention and control groups’ children engaged in 8076 and 7976 pedometer step-counts, respectively. There were not significant differences between the intervention and control groups across demographic characteristics, anthropometric measurements and pedometer step-counts at baseline.

### 3.2. Intervention Effects: Between Groups Differences

Comparison between the groups in anthropometric measurements and pedometer step-counts over the nine months are presented in Table 2. Children in the intervention group showed significantly lower BAZ (weighted difference: −0.12; 95% CI: −0.21, −0.03; *p* = 0.009), body fat percentage (weighted difference: −2.6%; 95% CI: −3.7, −1.5; *p* < 0.001) and waist circumference (weighted difference: −2.4 cm; 95% CI: −3.8, −1.0; *p* = 0.001) compared to the control group. On the other hand, no significant difference in pedometer step-counts was found between the intervention and control groups.

### 3.3. Intervention Effects: Within Groups Differences

Nine-month changes in anthropometric measurements and pedometer step-counts within-group are demonstrated in Table 3. Children in the intervention group reported significantly lower body fat percentage (weighted difference: −3.4%; 95% CI: 1.8, 5.0; *p* < 0.001) and waist circumference (weighted difference: −2.1 cm; 95% CI: −3.7, −0.5; *p* = 0.014) at T1 compared to baseline T0. However, the same group of children failed to produce significant change in BAZ (weighted difference: −0.07; 95% CI: −0.15, 0.01; *p* = 0.092) and pedometer step-counts (weighted difference: −8; 95% CI: −60, 45; *p* = 0.812) but a tendency towards lower BAZ and pedometer step-counts over the thirteen weeks. At T2, children in the intervention group showed tendency toward higher BAZ, body fat percentage, waist circumference and pedometer step-counts within T1 and T2. Apart from the above tendency, there was no significant changes for all the anthropometric measurements in intervention group’s children within T1 and T2, as well as T0 and T2. However, children in the intervention group had lower BAZ, body fat percentage and waist circumference but higher pedometer step-counts at T2 compared to T0.

Whereas children in the control group did not show any significant changes in all the anthropometric measurements and pedometer step-counts within T0 and T1 but a tendency towards higher anthropometric measurements and pedometer step-counts over the thirteen weeks. The same group children showed significantly higher BAZ (weighted difference: 0.10; 95% CI: −0.05, 0.16; *p* = 0.001), body fat percentage (weighted difference: 1.7%; 95% CI: 0.7, 2.7; *p* = 0.001) and waist circumference (weighted difference: 1.8 cm; 95% CI: 0.7, 3.0; *p* = 0.002) at T1 compared to T2 but no significant changes was observed for pedometer step-counts within T1 and T2. Similar outcomes were reported within T0 and T2, where children in control group showed significantly higher BAZ (weighted difference: 0.18; 95% CI: 0.10, 0.26; *p* < 0.001), body fat percentage (weighted difference: 2.2%; 95% CI: 1.3, 3.0; *p* < 0.001) and waist circumference (weighted difference: 2.5 cm; 95% CI: 0.9, 4.1; *p* = 0.002) at T2 compared to T0 but no significant changes was found for pedometer step-counts within T0 and T2.

## 4. Discussion

To our knowledge, this is the first reported multi-component intervention designed to test the feasibility of increasing whole grains intake as a strategy to manage childhood obesity. Outcomes from this feasibility trial involving multi-component whole grains with a healthy balanced diet intervention are suggestive of reducing anthropometric measurements independently of changes in physical activity level. Given that the prevalence of childhood obesity in Malaysia is high [3], these positive findings have the potential to impact the long-term management of childhood obesity in Malaysia.

A majority of the children belonged to the medium household income family, hence, the selected examples of wholegrain foods which were supplied to the children were considered appropriate and cost-effective [31]. The average pedometer step-counts for intervention and control group were 8076 and 7976, respectively. The results suggested that children from the present trial were less active than children from several European countries [32]. The observed lack of physical activity among overweight/obese children compared to normal weight children from the European countries may be attributed to the lower pedometer step-counts in the present trial. Despite offering free wholegrain foods on a daily basis during school break time, only 80% (n = 32) of the children in intervention group remained at T1. And of these, 85% of children attended at least five of six education classes. This indicates that the 15% drop out rate at T1 was solely due to lack of acceptance of wholegrain foods. Wholegrain food is consumed by only a minority of Malaysian children [20], thus it is imperative that collaborative efforts to investigate the factors influencing whole grains intake in Malaysian children is needed. There were no significant differences between the intervention and control groups across demographic characteristics, anthropometric measurements and pedometer step-counts in the baseline data. It is in accordance with that criteria suggested by a previous study, in which the intervention and control groups should have similar baseline data [33].

Children in the intervention group showed significantly lower BMI-for-age *z*-score (BAZ), body fat percentage and waist circumference compared to control group over the nine months. These findings are well-supported by three whole grains randomized intervention trials conducted among adults [34,35,36]. A number of possible mechanisms may underlie our findings in which whole grains consumption decreased the anthropometric measurements among children in the intervention group. Whole grains contain germ, bran and endosperm: which are a good source of B vitamins, fiber and minerals [15]. Further, whole grains contain physico-chemical properties and have lower energy density, which is good in weight management [15]. Consumption of whole grains may result in a delayed absorption and digestion of its starch and thus result in relatively lower postprandial insulin and glucose responses; lower insulin response may result in the lipolysis and oxidation of fat rather than its storage, thereby reducing body weight and body fat percentage [37]. Additionally, the overall lower glycemic index and high fiber content in whole grains may increase satiety and result in decreased calorie intake and body weight [15].

On the other hand, no significant difference in pedometer step-counts was found between intervention and control groups. This outcome is very likely attributed to the components of GReat-Child Trial, where the physical activity segment was relatively small; only involving simple verbal advice regarding physical activity for parents during an hour long individual diet counselling, without aerobic or physical activity session for the children. According to a systematic review, interventions which aimed to improve the physical activity level in children should be regarded as a simple, non-expensive and enjoyable way to reach all the children with adequate doses of moderate to vigorous physical activity [38].

BAZ is the most widely used tool to determine children’s nutritional outcome. Growth curves giving body mass index (BMI) distribution as a function of age and sex have been created to ensure more adoption of this tool in the children population [39]. We found BAZ in the intervention group did not show any significant differences over the 12-week intervention and six-month follow up and the changes were much lower compared to a childhood obesity intervention in Australia, which focused on dietary modification (weighted difference: −0.39; 95% CI: −0.51, 0.27) [40]. In contrast to this finding, several cross-sectional studies reported that a high intake of wholegrain foods had consistently demonstrated a significant inverse association with BAZ [14,17]. However, BAZ outcome from the present study is similar with the outcome from a Cochrane review [41], where childhood obesity interventions which focused on dietary modification alone did not significantly decrease BAZ (weighted difference: 0.06; 95% CI: −0.21, −0.01). This discrepancy is very likely attributed to differences in selected population, study designs, total calorie intake, specific type of dietary modification and the type of wholegrain foods consumed [42]. According to a systematic review, short-term childhood obesity interventions (less than 12-month) focused on dietary approach did not significantly decrease BAZ [43], nonetheless, these interventions had shown a positive effect on either blood sugar profile, lipid profile, systematic blood pressure or metabolic health, without significant changes in BAZ [44,45,46,47,48,49].

Body fat percentage decreased significantly at T1 in the intervention group, it is in accordance with that from a systematic review, where whole grains interventions did not show any effect on body weight but had an effect on the body fat percentage (weighted difference: −0.48%; 95% CI: −0.95, −0.01) [42]. The health benefits of weight loss are usually associated with the advantages of losing body fat mass. Body fat percentage may be a more sensitive measure compared to BAZ, because BAZ does not distinguish between fat and fat free mass but body fat percentage only includes fat mass [42]. On the other hand, the body fat percentage finding in the present study is in contrast with that from a childhood obesity intervention in United Kingdom named MEND (Mind, Exercise, Nutrition, Do It!), which is a family-based community intervention, focused on dietary modification to manage childhood obesity [50]. In which MEND did not show significant changes in body fat percentage. According to Hunt et al. (2007) [51], BAZ needs to fall by at least 0.5 to be relatively certain of definite body fat percentage reduction. In the present study, the significant decrease of body fat percentage without 0.5 reduction in BAZ might be a result of whole grains component in our 12-week program; in contrast to the MEND intervention, which consisted of a shorter program with 18 sessions delivered over 9 weeks. Their sessions comprised of an introduction meeting, 8 sessions focusing on behavior change, 8 sessions providing nutrition education, 16 physical activity sessions and a closing session. Further, MEND intervention did not emphasize whole grains in their nutrition educations.

Waist circumference is known to have greater measurement error and variability over time compared to other anthropometric measurements [52]; however, convincing evidence indicates that waist circumference was a better predictor for cardiovascular diseases in young children [53]. Its measurement is being encouraged to better assess effectiveness of childhood obesity intervention [54]. Waist circumference decreased significantly at T1 in intervention group, comparing favorably with the results reported by two other randomized studies of multidisciplinary lifestyle intervention [46,55] and pharmaceutical management intervention [56,57] for childhood obesity.

There were no statistically significant differences for physical activity within the two groups over the 12-week post-intervention and 6-month follow-up. A possible explanation for the minimal change in physical activity could be that physical activity was not greatly emphasized and not sufficiently intense in the current intervention [58], as this present trial emphasized on whole grain with healthy balanced diet intervention. The physical activity outcome of the present trial is well-supported by two systematic reviews which investigated the effectiveness of physical activity interventions in children [59,60]. Physical activity interventions, on average, achieved small to negligible increases in children’s total activity volume [60]. Potential factors that may have limited the effectiveness are the levels of exposure to the physical activity intervention and adherence [61].

Sustainability of results is crucial in assessing weight-management interventions [50]. In the present study, T1 outcomes did not show favorable sustainable effects at T2 and this is well-supported by previous studies [62,63]. Childhood obesity intervention is often focused on individual lifestyle changes including dietary modification, this approach may result in short-term behavior changes, yet evidence suggested that this approach may not have significant or sustainable impact [62]. Possible explanations for unfavorable sustainable effects could be due to either small sample sizes or follow ups that were too short or lack of control groups [63]. This is a quasi-experimental trial and that it is unknown what exactly true consumption and dietary practices of the schoolchildren in the present trial. In addition, the parents and children’s expectations and demand for wholegrains food to be supplied at school for free as a long-term basis was perceived as a barrier to the sustainability of the present trial. Parental involvement is a critical and feasible component in the present study which raises positive dietary behavioral changes for children in increasing the availability of wholegrain food at home.

However, anthropometric measurements in the intervention group appeared to be lower at T2 compared to T0. Evidence showed that overweight/obese children tended to have faster and higher increment rate in anthropometric measurements compared to normal weight children [50]. Despite T1 outcomes did not show favorable sustainable effects at T2, it helped in slowing down the increment rate in all the anthropometric measurements. Our trial should be considered in light of several limitations. One such limitation, present in almost all childhood obesity interventions was that the follow-up period was relatively short, which limits conclusions about the long-term effects of the intervention. To address this limitation, a follow-up of the intervention to investigate the sustainability of the childhood obesity interventions in Malaysia is currently in progress [64]. Intervention components in the GReat-Child Trial did not include physical activity that was sufficiently intense, which might be a result of this present trial emphasizing on whole grain with healthy balanced diet intervention instead. To minimize this limitation, simple verbal advice on physical activities was given to the parents during an hour of individual diet counselling. However, this might have limited impact on the children.

Despite these limitations, the present trial has several strengths worth noting. The GReat-Child Trial represented a novel approach to synthesize and profile the recent literature on childhood obesity interventions to examine the effectiveness of intervention with whole grain and healthy balanced diet in managing childhood obesity. Social cognitive theory was used in a previous study to improve whole grain intake; however, this theory has never been applied in childhood whole grain intervention and thus this study is the first of its kind to use this theory in its intervention. Development of a multi-component intervention based on the social cognitive theory, in which successfully improved whole grains intake among the children in a previous intervention [22]. Household income was controlled as covariate, which previously had been shown to be amenable to whole grain consumption in Malaysian children [20].

## 5. Conclusions

In conclusion, the GReat-Child Trial has provided important information regarding the effectiveness of whole grain with balanced diet intervention in managing childhood obesity. On the whole, the findings of the present trial demonstrate that increasing whole grains intake as a strategy to manage childhood obesity may significantly reduce body fat percentage and waist circumference independently of changes in physical activity level. Children in the intervention group showed significantly lower BAZ, body fat percentage and waist circumference compared to control group over the nine months. Dropout rate at post-intervention was mainly due to wholegrain foods preferences, in which it appears to be a population-wide concern. Empowering parents to play an equal role in intervention design and implementation is a promising approach to manage childhood obesity. We anticipate the GReat Child trial to be a pioneer that could be implemented by the government and policy makers to increase whole grain consumption among Malaysian children and to be incorporated into childhood obesity intervention programs.

## Figures and Tables

**Table 1:** Baseline demographic characteristic, anthropometric measurements and pedometer step-count in the GReat-Child Trial.

Variables	Intervention; n (%) (n = 31)	Control; n (%) (n = 32)	*p-*Value
Age; mean (SD)	10.66 (0.60)	10.63 (0.63)	0.882 ^†^
Sex			0.262 ^††^
Boys	18 (58.1)	15 (46.9)	
Girls	13 (41.9)	17 (53.1)	
Household income; mean (SD)	4506.45 (2384.94)	3612.50 (1054.56)	0.058 ^†^
Low (<RM2300)	3(9.7)	3 (9.4)	0.452 ^††^
Medium (RM2300–RM5599)	23 (74.2)	27 (84.4)	
High (≥RM5600)	5 (16.1)	2 (6.2)	
Anthropometric measurements			
BMI-for-age *z*-score; mean (SD)	2.35 (0.98)	2.12 (0.81)	0.324 ^†^
Body fat percentage (%); mean (SD)	36.9 (11.9)	35.6 (9.6)	0.145 ^†^
Waist circumference (cm); mean (SD)	79.5 (11.9)	75.6 (9.9)	0.166 ^†^
Pedometer steps; mean (SD) ^¥^	8076 (739)	7970 (658)	0.419 ^†^

^†^ Independent *T* test; ^††^ Chi-Square test; SD: standard deviation; ^¥^ involved 55 children (28 from intervention group and 27 from control group).

**Table 2:** Comparison between groups in all outcome measures over the nine months (*n* = 63).

	Intervention Group-Control Group	
	Mean (95% CI)	*p*-Value
BMI-for-age z-score ^†^	−0.12 (−0.21, −0.03)	0.009 **
Body fat percentage (%) ^††^	−2.6 (−3.7, −1.5)	<0.001 ***
Waist circumference (cm) ^†††^	−2.4 (−3.8, −1.0)	0.001 **
Pedometer step-count ^¥,††††^	−4 (−73, 66)	0.919

^†^ F-stat(df) = 7.35(1), *p-*value = 0.009; ^††^ F-stat(df) = 23.6(1), *p-*value < 0.001; ^†††^ F-stat(df) = 11.4(1), *p-*value = 0.001; ^††††^ F-stat(df) = 0.01(1), *p-*value = 0.919; Repeated measures ANCOVA between group analysis was applied followed by pairwise comparison; Household income and baseline variables were controlled by using repeated measures ANCOVA; ^¥^ involved 55 children (28 from intervention group and 27 from control group); ** significant at the 0.01 level; *** significant at the 0.001 level.

**Table 3:** Nine-month changes in all outcome measures within-group (n = 63).

		Intervention Group		Control Group	
	Comparison	Mean (95% CI)	*p*-Value	Mean (95% CI)	*p*-Value
BMI-for-age *z*-score	T1-T0	−0.07 (−0.15, 0.01)	0.092	0.07 (0.01, 0.14)	0.032
	T2-T0	−0.06 (−0.25, 0.13)	0.544	0.18 (0.10, 0.26)	<0.001 ***
	T2-T1	0.01 (−0.17, 0.19)	0.905	0.10 (0.05, 0.16)	0.001 **
Body fat percentage (%)	T1-T0	−3.4 (1.8, 5.0)	<0.001 **	0.4 (−0.1, 0.9)	0.081
	T2-T0	−1.6 (−3.8, 0.6)	0.154	2.2 (1.3, 3.0)	<0.001 ***
	T2-T1	1.8 (−0.5, 4.2)	0.127	1.7 (0.7, 2.7)	0.001 **
Waist circumference (cm)	T1-T0	−2.1 (−3.7, −0.5)	0.014 *	0.7 (−0.3, 1.7)	0.165
	T2-T0	−1.9 (−4.1, 0.3)	0.091	2.5 (0.9, 4.1)	0.002 **
	T2-T1	0.2(−1.5, 1.8)	0.812	1.8 (0.7, 3.0)	0.002 **
Pedometer step-count ^¥^	T1-T0	−8 (−60, 45)	0.768	30 (−18, 78)	0.212
	T2-T0	35 (−31, 100)	0.290	51 (−83, 184)	0.440
	T2-T1	42 (−7, 91)	0.087	−21 (−109, 150)	0.742

T0—Baseline; T1—post intervention (thirteenth week); T2—follow up (ninth month); Repeated measures ANCOVA within group analysis was applied followed by pairwise comparison with confidence interval adjustment; Household income and baseline variables were controlled by using repeated measures ANCOVA; ^¥^ involved 55 children (28 from intervention group and 27 from control group); * significant at the 0.05 level; ** significant at the 0.01 level; *** significant at the 0.001 level.
